# Performance of neck circumference to predict obesity and metabolic syndrome among adult Saudis: a cross-sectional study

**DOI:** 10.1186/s40608-019-0235-7

**Published:** 2019-04-01

**Authors:** Rasmieh Alzeidan, Amel Fayed, Ahmed S. Hersi, Hala Elmorshedy

**Affiliations:** 1 Cardiac Sciences Department Riyadh, King Saud University, College of Medicine, Riyadh, Saudi Arabia; 2 College of Medicine, Princess Nourah bint Abdulrahman University, Riyadh, Saudi Arabia; 3 High Institute of Public Health, Alexandria University, Alexandria, Egypt

**Keywords:** Central obesity, General obesity, Metabolic syndrome, Neck circumference, Saudi Arabia

## Abstract

**Background:**

Neck circumference (NC) is a novel simple and stable body measurement, a growing body of evidence indicates its validity to diagnose obesity and metabolic syndrome (MetS). Because the cutoff value of NC is gender and ethnic-specific; we conducted the current study to explore the performance of NC to predict general obesity, central obesity, and MetS among adult Saudis of both genders.

**Methods:**

This is a cross-sectional study which included 3063 adult Saudis (1156 males and 1907 females) with a mean age of 38.6 ± 14.1 years. Anthropometric measurements and blood pressure were assessed by a standardized methodology. Blood tests including fasting lipid panel, blood glucose, fasting blood glucose and hemoglobin A1c (HBA1c) were measured for all participants. We identified the MetS based on Adult Treatment Panel III (ATPIII definition). Data were analyzed using SPSS®19 (PASW statistics data document 19); NC was compared to relevant anthropometric measures to predict obesity and MetS using Receiver Operator Characteristic (ROC) analyses. The cutoff value of NC which possessed good discriminating power between obese and non-obese patients was estimated by Youden index, and we estimated the adjusted Odds Ratio (OR) to delineate the association between NC and the outcome variables by multiple logistic regression analysis.

**Results:**

ROC analyses demonstrated good performance of NC for general obesity, central obesity and MetS; as a predictor of obesity in non-diabetics, Area Under the Curve (AUC) ranged from 0.77–0.86. In MetS, AUC was 0.77 and 0.82 for males and females respectively. The best cutoff values of the NC to predict obesity were ≥ 37.5 cm for males versus ≥32.5 cm for females. The results of adjusted logistic regression analysis adjusted for age and waist height ratio, revealed a consistent positive association between NC, general obesity, MetS, and central obesity: ORs were 4.26, 3.03, 1.45 for males versus 4.65, 3.66, and1.47 for females respectively.

**Conclusion:**

NC stands out as an independent predictor of obesity and the MetS. Its stability, easiness of application, low cost and the cultural acceptance, justify its use as a screening tool for general and central obesity as well as MetS among Saudis under community settings, and as an additional routine measurement for health professionals.

## Background

The epidemic of obesity constitutes a world-wide major health problem; its consequences on the occurrence of cardiovascular diseases, type 2 diabetes are major concerns for physicians because of the associated poor quality of life, increased morbidity and mortality [1–5].

In the Kingdom of Saudi Arabia (KSA), the economic transition moving toward more wealth and prosperity has increased the risk of obesity through multiple interplaying factors including changes in dietary habits, and in behavioral factors associated with sedentary lifestyles [6]. Such tremendous lifestyle changes seemed to have fueled the obesity epidemic in the country and resulted in the most rapidly increasing prevalence of obesity in the world [7]. This phenomenon matches the situation observed in other Gulf Cooperation Council (GCC) countries, as well as many other Arab countries [8, 9]. Hence continuous monitoring of obesity is essential to estimate the magnitude of the problem, allocate resources and implement effective interventions.

Validated measures of obesity and body fat include; Body mass index (BMI), the waist-height ratio (WHtR), waist-hip ratio (WHpR), and waist circumference (WC). Except for WC, all of them are computed based on more than one physical body measurements, and the last three should be measured on light clothing, which could be cumbersome in cold weather. Blood tests which measure dyslipidemia, and determine the risks of cardiovascular diseases are invasive and expensive [10]. Advanced highly sensitive diagnostic tools to assess body fat as computed tomography (CT), and magnetic resonance imaging (MRI) are available, yet their application is limited for clinical settings since they are quite costly, and troublesome [11].

Neck Circumference (NC) emerges as a novel, simple, and discrete upper body measurements which differentiates between obese and non-obese. Moreover, several studies demonstrated the validity of NC as a measure of metabolic syndrome (MetS), as it correlates positively with the classical anthropometric indices such as BMI, WC, and WHtR [12–14]. NC cutoff values varies according to age, gender and ethnic group. For example: among Brazilian adolescence, the cutoff values of NC among overweight boys and girls were 31.25, and 34.25 cm, and were 32.65 and 37.95 cm, among obese boys and girls respectively [12].Considering variations due to ethnicity; the cutoff for NC among Chinese adolescents is lower than that for the Brazilian adolescents [15].

As a measure of upper body subcutaneous fat (UBSF), NC exhibits a higher metabolic risk than lower subcutaneous fat, and abdominal visceral fat, it correlates positively with free fatty acids, insulin resistance, very-low-density lipoprotein cholesterol (VLDL-C) production, oxidative stress, endothelial cell dysfunction, hypertension and vascular injury [16–18]. When both neck and abdominal visceral fat are elevated, they increased the risk of arterial stiffness and dyslipidemia as compared to elevated abdominal visceral fat only [14].

Despite the advantages of NC as a simple, cheap, stable throughout the day, more feasible in cold weathers, and more acceptable in conservative communities -Middle East, Gulf countries-, also, it is more appropriate in morbidly obese, where the abdominal belly hinders accurate measurements of waist and hip circumferences, nevertheless, few studies have investigated its performance in Saudis. In addition, one of the shortcomings of a recent study is that the authors relied on reported risk factors to evaluate the performance of NC to predict cardiometabolic risks [19]. Hence, in the present study, we aim to explore the utility and applicability of NC to predict general and central obesity and MetS in a representative sample of adult Saudis of both genders using quantitative measures according to international guidelines.

## Methods

The current study is a continuation of the cross-sectional Heart Health Promotion (HHP) study published previously [20]. HHP was conducted in Riyadh, Saudi Arabia, between 2013 and 2014 amongst King Saud University (KSU) employees and their families.

The initial study was approved by the Institutional Review Board (IRB) of the College of Medicine, University of King Saud (reference number 13–3721), and the study was conducted in accordance with the guidelines in the Declaration of Helsinki. All participants gave informed consent prior to enrollment in the study. Details of participants’ recruitment and their characteristics have been described earlier [20].

Data were collected using a modified form of the “WHO STEPwise approach to chronic disease risk factor surveillance- Instrument-V2.1” (both Arabic and English Forms). This instrument uses sequential steps which include: questionnaire (Step1), anthropometric measurements (Step II), and biochemical measurements (Step III) [21].

### Study sample

In this study, non-Saudis were excluded; the total sample size amounted to 3063 individuals of both genders. Considering obesity proportion of 25% ± 0.05, *p* < 0.01, the power of the study using STATA/IC14.2 was > 0.9.

### Anthropometric measurements

NC was measured for all Participants, using a non-stretch Teflon, with increments down to 0.1 cm. Participants were asked to stand erect with their head positioned at Frankfort horizontal plane. The superior border of a tape measure was placed just below the laryngeal prominence and applied perpendicular to the long axis of the neck. NC was obtained at a point just below the larynx (Adam’s apple) and perpendicular to the long axis of the neck [22, 23].

Valid measures of central obesity including WC, hip circumference (HC), WHpR and WHtR were estimated according to standardized techniques. The cutoff values used to diagnose central obesity based on WC, and WHpR were: ≥102 cm, and ≥ 0.9 for males, and ≥ 88 cm, and 0.85 for females respectively [24–26]. While the cutoff value of WHtR was ≥0.5 for both genders [26].

BMI is defined as a measure of general obesity and was calculated as the ratio between weight (kg) and squared height (m^2^) Height was measured without shoes to the nearest 0.01 m on a stadiometer, and weight in kilogram was measured in light clothing on a level balance to the nearest 0.01 kg. The balance was checked for accuracy at frequent intervals. Obesity was defined as BMI ≥ 30 kg/m^2^ [27].

### Definition of cardiometabolic risk factors

#### Hypertension

Participants were deemed hypertensive if they self- reported that they were currently using any anti-hypertensive medications regardless of their blood pressure readings. Participants were subsequently classified, as per the guideline in “The Seventh Report of the Joint National Committee (JNC7)” [28].

#### Diabetes mellitus

Participants were identified as having diabetes (type I or type II) if they reported current use of any diabetes medications, or reported a previous diagnosis of diabetes, or if their blood tests in this study showed any increase in a value HBA1C. Based on WHO and American Diabetes Association (ADA) criteria, participants were considered as having diabetes if the HBA1C level was ≥6.5% [29, 30].

#### Dyslipidemia

Subjects were categorized as having any sort of dyslipidemia according to the WHO and the Third Adult Treatment Panel (ATP-III) of the National Cholesterol Education Program (NCEP). Dyslipidemia includes raised level of TC, and/or LDL-C, or/and TGs and low level of HDLC, or if the subject reported using medications to lower blood lipid levels [24, 31].

All Participants were asked to fast 12 h before undergoing for a blood draw to measure lipid profile and fasting plasma glucose. Also, HBA1C was performed to all participants.

#### Metabolic syndrome (MetS)

Participants were identified as having MetS according to the NCEP-ATPIII criteria which relies on at least three out of five factors namely; central obesity, elevated triglycerides, reduced HDL-Cholesterol (HDL-C), elevated blood pressure, and elevated fasting glucose [24].

#### Statistical analysis

Data were analyzed using SPSS®19 (PASW statistics data document 19). Categorical data were summarized as frequency and percentages and were compared using Chi-square Goodness of fit test, while numeric data were summarized as means and standard deviations (SD), and were compared using independent sample t-test or Mann-Whitney-U test after testing for normality.

Receiver operating characteristic (ROC) analyses by gender were used to assess the accuracy of NC to predict general obesity, central obesity, and MetS in reference to valid indices (BMI as the gold slandered for general obesity, WHpR as the gold standard for central obesity, and NCEP-ATPIII criteria as the gold standard for MetS). Gender-specific NC cutoff values were estimated using the maximum value of the Youden’s index which is defined as: “sensitivity + specificity -1” [13].

Odds ratio with 95% CI were determined by multiple logistic regression analyses in models adjusted for age and WHtR as they provided the best model fit for the data. A *P*-value < 0.05 was considered statistically significant.

## Results

This study encompassed 3063 participants with a mean age of 38.6 ± 14.1 years, a minimum of 18, and a maximum of 85 years. Table 1 depicts the characteristics of the study sample by gender, Female to male ratio was 1.65, while their mean ages were comparable. Males had statistically higher values for the majority of anthropometric measurements, except the mean value of BMI was higher in females, and the proportion of obese females was 39.2% versus 33.1% in males. Males had statistically higher mean values than females for systolic blood pressure, diastolic blood pressure, most of the lipid profile, but the total cholesterol level and, the fasting blood glucose levels were statistically equal in both genders. More than two-thirds of males, and half of females had dyslipidemia, the difference was statistically significant. The results revealed no statistical difference in the proportions of diabetes, hypertension and MetS between genders.

**Table 1:** Characteristics of the Study Sample according to Gender

	Males (*n* = 1156)	Females (*n* = 1907)	*P* value
Variables	Mean ± SD	Mean ± SD	
Age in years	38.00 ± 14.34	39.00 ± 13.94	0.068
Neck Circumference(cm)	37.87 ± 3.05	33.35 ± 3.05	< 0.001
Waist Circumference (cm)	90.20 ± 14.58	79.51 ± 13.41	< 0.001
Hip Circumference (cm)	104.29 ± 11.66	106.25 ± 12.21	< 0.001
Waist/Height Ratio	0.53 ± 0.09	0.50 ± 0.09	< 0.001
Waist/Hip Ratio	0.86 ± 0.09	0.75 ± 0.15	< 0.001
BMI, kg/m^2^	28.20 ± 5.99	28.68 ± 6.41	0.047
SBP, mm Hg (average of 2 readings)^	122.00,17.00	114.00, 20.00	< 0.001
DBP, mm Hg (average of 2 readings)	73.70 ± 12.78	67.99 ± 8.93	< 0.001
LDL, mmol/L	3.11 ± 0.89	3.02 ± 0.80	0.003
TC, mmol/L	4.87 ± 0.97	4.92 ± 0.91	0.176
TG, mmol/L	1.42 ± 1.08	1.09 ± 0.65	< 0.001
HDL, mmol/L	1.13 ± 0.29	1.40 ± 0.36	< 0.001
Blood Glucose, mmol/L^	4.70, 1.30	4.70, 1.00	0.101
Obesity (BM1 ≥ 30 kg/m^2^) (n,%)	283 (33.10)	747 (39.20)	0.001
Dyslipidemia (n,%)	805 (70.40)	1335 (53.20)	< 0.001
Diabetes Positives (n,%)	200 (17.30)	349 (18.30)	0.484
Hypertension Positives (n,%)	252 (21.80)	361 (18.90)	0.054
Metabolic Syndrome (n,%)	249 (21.50)	384 (20.10)	0.352

Legend: *SD* Standard Deviation, *n* number, ^ skewed variables presented in median, Interquartile range and used Mann-Whitney test for comparison

*BMI* body mass index, *SBP* systolic blood pressure, *DBP* diastolic blood pressure, *TC* total cholesterol, *HDL* high density lipoprotein cholesterol, *LDL* low density lipoprotein Cholesterol, *TAG* triglycerides

In Table 2; we compared the performance of NC versus the other anthropometric indices according to gender, and diabetic status using ROC analyses to diagnose general obesity as based on BMI, and central obesity as based on WHpR according to WHO guidelines [32]. The AUCs of neck circumference possessed a good discriminating ability for general obesity, its value ranged from 0.82 to 0.84. But, the performance of WC and WHtR exceeds that of NC as their computed AUCs ranged from 0.91 to 0.93. Among diabetics, the performance of all the aforementioned indices was slightly attenuated. On whole, all indices had a slightly better performance among males. Of note, the WHpR ratio which measures central obesity, had the lowest values of AUCs as compared to other indices, furthermore, it failed to discriminate between obese and non-obese in diabetics.

**Table 2:** Area under the Curve (AUC) by Anthropometric Indices for General, and Central Obesity among Adult Saudis according to Gender, and Diabetic Status

	AUC (CI 95%)			
	NC	WC	WHtR	WHpR
Obesity (Diabetics and non-diabetics)				
Males (1156)	0.84 (0.82–0.87)	0.92 (0.90–0.93)	0.92 (0.90–0.94)	0.70 (0.67–0.73)
Females (1907)	0.83 (0.81–0.85)	0.91 (0.89–0.92)	0.91 (0.90–0.92)	0.69 (0.67–0.72)
Obesity (Non-diabetics)				
Males (956)	0.86 (0.83–0.88)	0.93 (0.91–0.95)	0.93 (0.91–0.95)	0.71 (0.68–0.75
Females (1556)	0.82 (0.80–0.84)	0.91 (0.90–0.92)	0.91 (0.90–0.93)	0.68 (0.66–0.71)
Obesity (Diabetics)				
Males (200)	0.77 (0.70–0.83)	0.88 (0.83–0.93)	0.91 (0.87–0.95)	0.62 (0.52–0.68)
Females (348)	0.77 (0.72–0.83)	0.84 (0.80–0.88)	0.85 (0.81–0.89)	0.52 (0.45–0.58)^
	NC	WC	WHtR	BMI
Central Obesity (Diabetics and Non-Diabetics)				
Males (1156)	0.80 (0.77–0.82)	0.88 (0.86–0.90)	0.89 (0.87–0.91)	0.75 (0.72–0.78)
Females (1907)	0.77 (0.74–0.80)	0.90 (0.88–0.92)	0.90 (0.82–0.92)	0.70 (0.67–0.73)
Central Obesity (Non-diabetics)				
Males (956)	0.79 (0.76–0.82)	0.88 (0.86–0.90)	0.89 (0.91–0.95)	0.72 (0.72–0.78)
Females (1556)	0.78 (0.74–0.82)	0.91 (0.88–0.93)	0.92 (0.89–0.94)	0.71 (0.67–0.75)
Central Obesity (Diabetics)				
Males (200)	0.71 (0.61–0.81)	0.81 (0.72–0.89)	0.82 (0.74–0.91)	0.64 (0.53–0.76)
Females (348)	0.60 (0.54–0.67)	0.78 (0.73–0.83)	0.78 (0.73–0.82)	0.50 (0.44–0.57)**^**

Legend: *NC* neck circumference, *WC* waist circumference, *WHtR* waist height ratio, *WHpR* waist hip ratio, *BMI* body mass index,^= not significant, *CI* Confidence Interval

Regarding central obesity, we included BMI which is used solely to estimate general obesity to highlight the difference between invalid and valid anthropometric indices. Overall, NC possessed a good discriminative ability, the AUCs were 0.80 and 0.77 for males and female respectively. However, the performance of WC and WHtR exceeds that of NC. Nevertheless, the discriminative ability of NC, WC and WHtR was lower than that for general obesity, with further attenuation in diabetics. Among all indices, NC was the only one which holds its consistency as a better measurement among males. As expected, BMI had the least discriminative ability, furthermore, it failed to discriminate between obese and non-obese in diabetics (Table 2).

In MetS, the AUCs of the NC equaled that of WHpR: 0.77, and 0.82 versus 0.77, and 0.83 for males, and females respectively. But the AUCs of WC and WHtR were slightly larger than that of NC. The AUC of BMI for males was the least of all indices (0.75) (Table 3).

**Table 3:** Area under the curve (AUC) by Anthropometric Indices for Metabolic Syndrome among Adult Saudis According to Gender

	AUC (95% CI)				
	NC	WC	WHtR	WHpR	BMI
Males (1156)	0.77 (0.74–0.81)	0.81 (0.78–0.84)	0.81 (0.78–0.84)	0.77 (0.74–0.80)	0.75 (0.72–0.78)
Females (1907)	0.82 (0.80–0.84)	0.88 (0.87–0.90)	0.89 (0.87–0.91	0.83 (0.81–0.85)	0.81 (0.79–0.84)

Legend: *NC* neck circumference, *WC* waist circumference, *WHtR* waist height ratio, *WHpR* waist hip ratio, *BMI* body mass index

We estimated the best cutoff values of NC to predict obesity and metabolic syndrome using Youden’s index. In males, the best cutoff value of NC was ≥37.5 cm, versus ≥32.5 cm for females. Using these cutoffs, both NPVs and sensitivities exceeded 85% across all categories; hence, we can rule-out obesity when the result is negative with a great precision. However, the value of NC to rule-in obese individuals was moderate according to the estimates of PPVs and specificities (Table 4).

**Table 4:** Neck Circumference Cutoff Values to Diagnose General Obesity in Adult Saudis According to Gender and Diabetic Status

	Sensitivity%	Specificity%	PPV%	NPV%
Total				
Male: N1156	86.4	64.2	54.4	90.5
Female: N 1907	88.9	61.3	59.7	89.5
Non-diabetics				
Male: N 956	84.9	69.4	55.8	91.0
Female: N 1558	85.5	65.1	54.8	90.0
Diabetics				
Male: N 200	91.7	34.5	50.3	85.1
Female: N 349	96.5	28.0	72.4	80.5

Legend: NC cutoff to diagnose obesity for males = 37.5 & for females is 32.5, *PPV* positive predictive value, *NPV* negative predictive value

Table 5 shows the results of logistic regression analysis, after adjusting for age, and WHtR. Results revealed consistent positive associations; the strength of association was the highest for general obesity, followed by MetS. In males, ORs were 4.26, and 3.03, which were analogous to ORs in females being 4.65, and 3.66 for obesity, and MetS respectively. Regarding central obesity, ORs were still positive- 1.47 and 1.45 for males and females respectively-, but the strength of association was weak, 95% CI was (1.02–2.08), and (0.95–2.26) for males and females respectively.

**Table 5:** Gender Specific Odds Ratio of Obesity and Metabolic Syndrome, for Neck Circumference among Adult Saudis

Variables	Adjusted OR	95% CI
General obesity		
Males	4.26	2.95–6.15
Females	4.65	3.43–6.29
Central Obesity		
Males	1.45	1.02–2.08
Females	1.47	0.95–2.26
Metabolic syndrome		
Males	3.03	2.95–6.15
Females	3.66	2.35–5.69

Legend: *OR* Odds Ration, Adjusted for age, and WHtR, *CI* confidence interval

## Discussion

We conducted the present study to explore the utility and applicability of NC to diagnose obesity and MetS among a segment of adult Saudis that represents the age group at high risk. The study encompassed a fairly large sample size amounted to 3063 individuals of both genders.

Our results demonstrated that the estimated cutoff values of NC for males and females were independently associated with general and central obesity, as well as with MetS after adjustment of relevant confounders. However, the strength of association was maximized for general obesity and MetS.

This study provides an additional indicator of the good performance of NC to predict general obesity in two ways. First, among non-diabetics, the AUCs of the ROC analyses ranged from 0.82–0.86. Second, the estimated neck cutoff values revealed high sensitivity and NPV across all categories of participants which ranged from 86 to 96%, and 80.5–91% for sensitivity and NPV respectively. Thus, we can rule-out obesity when the result is negative with a great precision. Accordingly, the utility of NC as a screening tool for general obesity is ascertained. In agreement with our results, several studies confirmed the validity of NC as a good measure of obesity, since it is correlated positively with WC, WHtR, and BMI [12–14].

In comparison to general obesity, the performance of NC to predict central obesity was slightly attenuated. This observation is expected because NC reflects UBSF [16–18]. Meanwhile, the attributes of the impaired diagnostic capability of NC as well as WC and WHtR in diabetics might be linked to the disease process which affects the dynamics of fat metabolism. However, further validation of NC among diabetics needs further investigation using a large sample size and a sensitive quantitative technique for the accurate estimation of body fat. In accordance with our results, several studies reported similar performance of NC to predict central obesity based on the ROC analyses which demonstrated values of AUCS comparable to our results [33–35].

Despite the fact that the performance of WC and WHtR exceeds that of NC to diagnose both types of obesity, yet NC might be efficient for several reasons. First, it might be more culturally acceptable in the Gulf region. Second, compared to WC and WHtR, its performance is stable between genders for both central and general obesity. Third, its stability is not affected by physical, or physiological conditions; in contrast, WC is affected by being full or hungry, respiratory movement, and wearing heavy clothing. In addition, the inter and intra-rater reliability of NC is high, with no need for multiple measurements [36] .

Regarding the MetS, as one might expect that WC, WHtR and WHpR as indices of visceral obesity would be better discriminators. Nevertheless, this study demonstrated that NC was comparable to WC and WHtR, and was almost identical to WHpR to predict MetS. Furthermore, a large Brazilian study confirmed that NC correlated positively with all known risk factors of MetS, and negatively with protective factors including insulin sensitivity and high-density lipids [37]. In addition, a recent study claimed that, in morbidly obese individuals, NC was even better than WC to assess the metabolic health [38].

We computed the NC cutoffs for both males and females which better discriminates between obese and non-obese. The dissimilarities of NC cutoff values between different studies; for example Chinese, Brazilian, and Turkish [13, 35, 37, 39] indicates that ethnic-specific cutoffs are essential, and it should be estimated based on large sample size to ensure generalizability of the study results. However a recent study from Saudi Arabia reported higher cutoff values in both mlaes and females; this variability might be related to the variations in sample size, characteristics of study participants and in the methodology used to determine the optimal cutoff; while we based our estimation on Youden’s index to determine the cutoff value for both general and central obesity [13]; in the aforementioned study, the authors reported the cutoff values for central obesity and it was not clear how the authors determined the selected values [19].

Finally, we used regression analysis to delineate the strength of association between NC, MetS and both general and central obesity for males and females separately after adjusting for age and WHtR. In both genders, ORs were very close, Therefore, we can exclude the effect modification due to gender. The strength of association was maximized for general obesity and MetS (OR > 3) which is more than double that for central obesity. Several studies considered NC as a novel single predictor of MetS [34, 35, 39]. Another Chinese study which included about 2000 adults of both genders concluded that NC has a power analogous to WC to identify metabolic disorders [40]. Furthermore, several studies confirmed that NC correlated positively with free fatty acids, insulin resistance, very-low-density lipoprotein cholesterol, oxidative stress, endothelial cell damage, hypertension and vascular injury, and was inversely correlated with HDL-C [16–18, 39]. In the present study, the uncertainty of NC to predict central obesity is logical, since NC is a measure of upper- body fat. This observation further confirms that NC predicts MetS beyond central obesity [41].

### Limitations

The convenient sampling technique and the cross-sectional study design might affect the generalizability of results. But, the large sample size with a power exceeding 0.9 and the completed documentations of all studied variables with no missing values could compensate for these limitations.

## Conclusion

ROC analyses demonstrated a good discriminative power of NC to predict obesity and metabolic syndrome. Furthermore, NC stands out as an independent predictor of general obesity and the MetS using cutoff values of ≥37.5 for men, and ≥ 32.5 for women. Its stability, ease of application, low cost and the cultural consideration justify its use as a screening tool for general obesity and MetS among Saudis under community settings, and as an additional routine measurement for health professionals.

## Abbreviations

ATP-III: Third Adult Treatment Panel
AUC: Area under the curve
BMI: Body mass index
CI: Confidence Interval
CT: Computed Tomography
CVDs: Cardiovascular diseases
CVRF: Cardiovascular risk factors
HbA1C: Glycosylated hemoglobin
HDL-C: High density lipoprotein cholesterol
LDL-C: low density lipoprotein cholesterol
MetS: Metabolic Syndrome
NC: Neck Circumference
NCEP: National Cholesterol Education Program
NPV: Negative predictive value
OR: Odds Ratio
PPV: Positive predictive value
ROC: Receiver operating characteristic curve
TC: Total cholesterol
TG: Triglycerides
WC: Waist circumference
WHpR: Waist hip ratio
WHtR: Waist Height Ratio
